# Molecular Identification of Broomrape Species from a Single Seed by High Resolution Melting Analysis

**DOI:** 10.3389/fpls.2016.01838

**Published:** 2016-12-12

**Authors:** Mathieu Rolland, Aurélie Dupuy, Aude Pelleray, Philippe Delavault

**Affiliations:** ^1^GEVES, BeaucouzéFrance; ^2^Laboratoire de Biologie et Pathologie Végétales, Université de NantesNantes, France

**Keywords:** *Orobanche*, *Phelipanche*, parasitic weed, molecular diagnosis, HRM, *trn*L, *rbc*L

## Abstract

Broomrapes are holoparasitic plants spreading through seeds. Each plant produces hundreds of thousands of seeds which remain viable in the soils for decades. To limit their spread, drastic measures are being taken and the contamination of a commercial seed lot by a single broomrape seed can lead to its rejection. Considering that broomrapes species identification from a single seed is extremely difficult even for trained botanists and that among all the described species, only a few are really noxious for the crops, numerous seed lots are rejected because of the contamination by seeds of non-noxious broomrape species. The aim of this study was to develop and evaluate a High Resolution Melting assay identifying the eight most noxious and common broomrape species (*Phelipanche aegyptiaca*, *Orobanche cernua*, *O. crenata, O. cumana*, *O. foetida*, *O. hederae*, *O. minor*, and *P. ramosa*) from a single seed. Based on *trn*L and *rbc*L plastidial genes amplification, the designed assay successfully identifies *O. cumana*, *O. cernua*, *O. crenata*, *O. minor*, *O. hederae*, and *O. foetida*; *P. ramosa*, and *P. aegyptiaca* can be differentiated from other species but not from each other. Tested on 50 seed lots, obtained results perfectly matched identifications performed by sequencing. Through the analysis of common seed lots by different analysts, the reproducibility of the assay was evaluated at 90%. Despite an original sample preparation process it was not possible to extract enough DNA from some seeds (10% of the samples). The described assay fulfills its objectives and allows an accurate identification of the targeted broomrape species. It can be used to identify contaminants in commercial seed lots or for any other purpose. The assay might be extended to vegetative material.

## Introduction

Broomrapes (*Orobanche* and *Phelipanche* spp.) are angiosperms in the Orobanchaceae which have evolved into obligate root holoparasitic plants (Joel, 2009). Devoid of leaves, of chlorophyll as well as of functional roots, they entirely depend on their host for nutritional requirements (Westwood, 2013). One single broomrape plant can produce hundreds of thousands of extremely small seeds, between 200 and 300 μm, each weighing around 5 μg and composed of only 200 to 300 cells (Joel, 1987). They are easily dispersed mainly by wind and water, and remain viable in the soils for many years until their germination is triggered chemically by exudates released in the soil by the roots of potential host plants (Lopez-Granados and Garcia-Torres, 1996, 1999). At the vicinity of host roots, germinated seeds develop a haustorium that penetrates host tissues and establish a connection with its vascular tissues. This connection will constitute the source of the parasite for water and nutrients. Some species in the *Orobanche* and *Phelipanche* genera, the weedy broomrapes, are able to infect a large range of plant species, including many important crops. In severe cases, infection of the host plant can lead in reduction of crop yields up 100%. This makes broomrapes one of the most devastating parasitic weeds in the Mediterranean and western Asian regions but also in many other parts of the world (Parker, 2009). Thus, among these noxious parasitic species are the closely related *Phelipanche ramosa* L. and *P. aegyptiaca* Pers. (synonym *Orobanche ramosa* and *O. aegyptiaca*) (Joel, 2009), *O. cumana* Wallr., *O. cernua* Loefl., *O. crenata* Forsk., *O. foetida* Poir., and *O. minor* Sm., while *O. hederae* Vaucher ex Duby has no agronomical impact but is extremely common.

Strategies to control parasitic weeds can be classified in chemical, cultural, physical, and biological control methods (Fernández-Aparicio et al., 2016). Among them, breeding for crop resistance seems to be the best approach to manage this issue. However, sources of resistance to most parasitic plants are either scarce or of complex nature (Perez-De-Luque et al., 2009). Despite these difficulties, significant success has been made on some crops. All these approaches allow a control of the parasitic population or permit resistant crops to grow and yield on infested soils, however, the eradication of parasitic weeds remains extremely difficult. Considering that one major mean of field contamination is through contaminated crop seed lots, preventive measures have to be taken to avoid spreading parasite seeds, especially through global scale seed exchanges. This requires to detect efficiently the possible contaminations of crop seeds lots by broomrape seed (Dongo et al., 2012). Visual detection of broomrape seeds in crop seed lots is conducted by sieving and observation of the obtained residues. Characterization of broomrape seeds at the species level in contaminated crop seed lots is important giving the differential host ranges among broomrape weed species and the capacity of some broomrape weeds to thrive in non-parasitic weed species. However, due to their nuanced microscopic morphological features, this identification is extremely difficult and can only can be performed by high qualified specialists (Abu Sbaih and Jury, 1994; Plaza et al., 2004). Molecular tools have been developed to detect and identify broomrape species from soil and crop seed batches. Random amplified polymorphic DNA technique (RAPD) allowed the differentiation between species such as *P. aegyptiaca*, *P. ramosa*, *O. cernua*, *O. cumana*, and *O. crenata* (Katzir et al., 1996; Paran et al., 1997). This technique was even used on single seeds (Portnoy et al., 1997), however, the main drawback of RAPDs is their low reproducibility (Harris, 1999). Intersimple sequence repeats (ISSR) were latter used to discriminate closely related species such as *O. cumana* and *O. cernua* (Benharrat et al., 2002). A TaqMan assay was developed on internal transcribed spacers (ITS) with the aim of detecting and quantifying *P. ramosa* and *O. cumana* seeds in oilseed rape and sunflower seed lots, respectively (Dongo et al., 2012). Microsatellites were also developed to investigate intraspecific variations in *O. cumana* (Pineda-Martos et al., 2014). Due to its monoparental inheritance, plastid genome has a low intraspecific variability and seems to be an adequate target for species identification. In the case of *Orobanche* genus, a particular attention was paid to the pseudogene *rbc*L which showed important sequence divergences among species due to an evolution under purifying selection (Wolfe and dePamphilis, 1997; Benharrat et al., 2000; Manen et al., 2004). Recently, full broomrape plastid genome sequence was made available (Wicke et al., 2013; Cusimano and Wicke, 2015) providing new molecular markers for species identification.

High resolution melting (HRM) is a technique based on the real-time measure of double stranded DNA denaturation at a high resolution. It is suitable for gene scanning and genotyping (Gori et al., 2012) and allows the detection of genetic variations such as single nucleotide polymorphisms (SNP), mutations (Toi and Dwyer, 2008), or methylation (Wojdacz and Dobrovic, 2007). Used on PCR products during a post-PCR denaturation, it requires no tube opening, purification, or product separation. With a minimum manipulation, HRM minimizes the contamination risk, it is cost efficient, suitable for high-throughput, and can be performed in-house by laboratories with no sequencing facility (Reed et al., 2007). This technique has been extensively used on human tissues (Krypuy et al., 2007; Takano et al., 2008), for clinical or phytopathological diagnostic and food analysis (Druml and Cichna-Markl, 2014). It is increasingly used on plant tissues for species and cultivar differentiation (Mackay et al., 2008; Jaakola et al., 2010) or genotyping (Lochlainn et al., 2011).

The objective of this study is to combine the knowledge recently obtained on plastid genome and the HRM technique to develop a new application allowing the differentiation of the seven most noxious and common broomrape weed species (*P. aegyptiaca*, *O. cernua*, *O. crenata, O. cumana*, *O. foetida*, *O. minor*, and *P. ramosa*) and the widely distributed *O. hederae* species from a single seed. This new application should provide to laboratories, involved in seed certification, a decision-making tool to evaluate crop seed lots potentially contaminated by noxious broomrape species.

## Materials and Methods

### Plant Material

Broomrape seeds (*P. aegyptiaca*, *O. cernua*, *O. crenata, O. cumana*, *O. foetida*, *O. hederae*, *O. minor*, and *P. ramosa*) were either obtained from international collections or collected during field sampling by GEVES, Syngenta, Terres Inovia, or University of Nantes. Available data concerning the tested seed lots are summarized in **Table 1**.

**Table 1:** Origin and identifications of the 50 seed lots tested during the study.

N°	Visual identification	Country (region)	Crop	Collector	Date	High resolution melting (HRM)			Sequencing
						trnL	rbcL	Identification	Identification
1	*P. ramosa*	Charente, France	Rape	LBPV	Unkn	trnL-C	NA	sub. ramosae	*P. ramosa*
2	*P. ramosa*	Aube, France	Hemp	LBPV	2011	trnL-C	NA	sub. ramosae	*P. ramosa*
3	*P. ramosa*	Bas-Rhin, France	Tobacco	LBPV	Unkn	trnL-C	NA	sub. ramosae	*P. ramosa*
4	*P. ramosa*	Vendée, France	Rape	LBPV	2009	trnL-C	NA	sub. ramosae	*P. ramosa*
5	*P. ramosa*	Charente-Maritime, France	Tobacco	LBPV	2012	trnL-B	rbcL-B	*O. minor*	*O. minor*
6	*P. ramosa*	Vendée, France	Rape	This study	2013	trnL- C	NA	sub. ramosae	*P. ramosa*
7	*P. ramosa*	Aube, France	Celery	This study	2013	NA	NA	NA	NA
8	*P. ramosa*	Charente-Maritime, France	Rape	This study	2014	trnL- C	NA	sub. ramosae	*P. ramosa*
9	*P. ramosa*	Vendée, France	Rape	LBPV	2011	trnL- C	NA	sub. ramosae	*P. ramosa*
10	*P. ramosa*	Aube, France	Hemp	LBPV	2007	trnL- C	NA	sub. ramosae	*P. ramosa*
11	*P. ramosa*	Unkn	Hemp	LBPV	2013	trnL- C	NA	sub. ramosae	*P. ramosa*
12	*P. aegyptiaca*	Israël	Unkn	LBPV	2011	NA	NA	NA	NA
13	*O. cumana*	Iazu, Romania	Sunflower	LBPV	Unkn	trnL- A	NA	*O. cumana*	*O. cumana*
14	*O. cumana*	Carmona, Spain	Sunflower	Authors	2014	trnL- A	NA	*O. cumana*	*O. cumana*
15	*O. cumana*	Haute-Garonne, France	Sunflower	Authors	2014	NA	NA	NA	NA
16	*O. cumana*	Vendée, France	Unkn	LBPV	2009	trnL- A	NA	*O. cumana*	*O. cumana*
17	*O. cumana*	Turcia, Turkey	Unkn	LBPV	Unkn	trnL- A	NA	*O. cumana*	*O. cumana*
18	*O. cernua*	Israël	Unkn	LBPV	Unkn	trnL- A bis	NA	*O. cernua*	*O. cernua*
19	*O. cernua*	Lucainena, Spain	Unkn	LBPV	2006	trnL- A bis	NA	*O. cernua*	*O. cernua*
20	*O. foetida*	Unkn	Chickpea	LBPV	Unkn	trnL- D	NA	*O. foetida*	*O. foetida*
21	*O. foetida*	Tunisia	Chickpea	LBPV	2014	trnL- D	NA	*O. foetida*	*O. foetida*
22	*O. minor*	Unkn	Unkn	Authors	1987	trnL- B	rbcL-B	*O. minor*	*O. minor*
23	*O. minor*	Vendée, France	Clover	Authors	2013	trnL- B	rbcL-B	*O. minor*	*O. minor*
24	*O. minor*	Utsunomiya, Japan	Unkn	LBPV	2013	trnL- B	rbcL-B	*O. minor*	*O. minor*
25	*O. minor*	Maine-et-Loire, France	Clover	Authors	2014	trnL- B	rbcL-B	*O. minor*	*O. minor*
26	*O. minor*	Côte-d’Or, France	Clover	Authors	2014	trnL- B	rbcL-B	*O. minor*	*O. minor*
27	*O. minor*	Nienberge, Germany	Unkn	Botanical garden of Munster	1995	trnL- B	rbcL-B	*O. minor*	*O. minor*
28	*O. minor* subsp. *maritima*	Unkn	Unkn	Authors	1987	trnL- B	rbcL-B	*O. minor*	*O. minor*
29	*O. crenata*	Meknes, Morocco	Peas	LBPV	2001	trnL- B	rbcL-A	*O. crenata*	*O. crenata*
30	*O. crenata*	Ariana, Tunisia	Unkn	LBPV	2007	trnL- B	rbcL-A	*O. crenata*	*O. crenata*
31	*O. crenata*	Unkn	Unkn	Botanical garden of Braunschweig	1994	NA	NA	NA	NA
32	*O. crenata*	Unkn	Unkn	Botanical garden of Zurich	1997	trnL- B	rbcL-A	*O. crenata*	*O. crenata*
33	*O. hederae*	Unkn	Unkn	Authors	1987	NA	NA	NA	NA
34	*O. hederae*	Maine-et-Loire, France	Climber	Authors	2012	trnL- B	rbcL-C	*O. hederae*	*O. hederae*
35	*O. hederae*	Maine-et-Loire, France	Climber	Authors	2012	trnL- B	rbcL-C	*O. hederae*	*O. hederae*
36	*O. hederae*	Maine-et-Loire, France	Climber	Authors	2014	trnL- B	rbcL-C	*O. hederae*	*O. hederae*
37	*O. hederae*	Finistère, France	Unkn	LBPV	1993	trnL- B	rbcL-C	*O. hederae*	*O. hederae*
38	*P. purpurea*	La palma, Spain	Unkn	Botanical garden of Zurich	Unkn	trnL- B	rbcL-C	*O. hederae*	*O. hederae*
39	*O. flava*	Unkn	*Aconitum variegatum*	Botanical garden of Zurich	Unkn	trnL- B	rbcL-C	*O. hederae*	*O. hederae*
40	*O. picridis*	Yvelines, France	Unkn	Authors	2014	trnL- B	NI	NI	minores spp
41	*O. artemisia campestris*	Maine-et-Loire, France	Unkn	Authors	2014	trnL- B	NI	NI	minores spp
42	*O. alsatica*	Binn, Switzerland	*Peucedanum cervaria*	Botanical garden of Zurich	Unkn	NI	NA	NI	NI
43	*O. alsatica*	Unkn	Unkn	Botanical garden of Frankfurt	2012	trnL-B	NI	NI	*O. bartlingii*
44	*P. arenaria*	Indre-et-Loire, France	Unkn	Botanical garden of Nantes	Unkn	NI	NA	NI	*P. arenaria*
45	*O. laevis*	Lax, Switzerland	*Artemisia campestris*	Botanical garden of Zurich	Unkn	NI	NA	NI	*P. arenaria*
46	*O. lucorum*	Unkn	*Berberis vulgaris*	Botanical garden of Zurich	Unkn	NI	NA	NI	*O. lucorum*
47	*O. gracilis*	Haute-Savoie, France	Unkn	Botanical garden of Alpin	Unkn	NI	NA	NI	*O. gracilis*
48	*O. caryophyllacea*	Binn, Switzerland	Galium spec.	Botanical garden of Zurich	Unkn	NI	NA	NI	NI
49	*P. mutelii*	Australia	Unkn	LBPV	Unkn	trnL-C	NA	sub. ramosae	*P. mutelii*
50	*P. mutelii*	Mannum south, Australia	Unkn	LBPV	2002	trnL- C	NA	sub. ramosae	*P. mutelii*

*NA, not amplified; NI, not identified; Unkn, unknown.*

### Single Seed Grinding Procedure and DNA Extraction

One of the technical challenges associated with the development of an assay aiming to characterize broomrape single seeds is the ability to obtain enough DNA from seeds weighing an average of 5 μg in a reproducible manner. To this end, each seed was crushed between two microscopy glass slides in presence of 2 μl of ultrapure water and seed tissues were then collected in a microtube. In order to maximize the amount of collected DNA, the slides were rinsed with 400 μl of PL1 extraction buffer (Macherey–Nagel) and the rinse collected in the microtube. Total DNA extraction was then performed using the NucleoSpin^®^ Plant II commercial kit (Macherey–Nagel) following the manufacturer’s instructions (filtration columns were not used). A control of the quantity and quality of the extracted DNA was performed using a NanoVue^TM^ Spectrophotometer (GE Healthcare).

### Sequencing

Thanks to previous studies on plastid genome sequences in broomrapes (Wicke et al., 2013; unpublished results), sequences corresponding to eight plastid genes (*rbc*L, *rps7*, *rps11*, *rpl36*, *rpl16*, *trn*Q, *trn*L, and *rrn23*) and one nuclear region (ITS) were obtained for the eight studied species. Sequences were aligned using the default alignment algorithm of Geneious v5.6.4. Two markers showing significant sequence divergence among the eight species were selected for subsequent HRM experiments: trnL and rbcL.

To design HRM primers and to control the identification of the species, pseudogenes *trn*L and *rbc*L were amplified and sequenced, respectively, using the primers (i) trnL C (F) and trnL HRM R, (ii) 1F and 1352R (**Table 2**). Amplification was performed on 5 μl of single seed total DNA extract, by 1 U of AmpliTaq Gold^®^ (Life Technologies), in a total volume of 40 μl at the final concentration of 1X of the appropriate Buffer II, 0.3 μM of each primer, 1.5 mM of MgCl_2_, and 0.2 mM of dNTP. PCR conditions were adjusted as follow, an initial denaturation of 10 min at 95°C, 40 cycles of 30 s at 95°C, 15 s at 58°C, and 1 min at 72°C, and a final extension of 10 min at 72°C. After migration in a 1.5% agarose gel at 180 V for 45 min and ethidium bromide staining, PCR products were visualized under UV light. Purification and sequencing of the PCR products was provided by Genoscreen.

**Table 2:** Primers used for amplification, sequencing, and HRM.

Target	Name	Sequence (5′–3′)	Source	Purpose
trnL	trnL C (F)	CGAAATCGGTAGACGCTACG	Taberlet et al., 1991	PCR and sequencing
	trnL HRM R	GGGGATAGAGGGACTTGAACC		
rbcL	1F	ATGTCACCACAAACAGAAAC	Manen et al., 2004	PCR and sequencing
	1352R	CAGCAACTAGTTCAGGRCTCC		
trnL	trnL-Z1-F	CGGTAGACGCTACGGACTTA	This study	HRM
	trnL-Lg-2R	ATGGGACTCTATCTTTATTCTC		
rbcL	rbcL-lg-1-F	AACCTGAAGTTCCGCCTGAA	This study	HRM
	rbcL-Z2-R	AGTACATCCCAACAGGGGAC		

### Primer Design

Obtained sequences were aligned using the default alignment algorithm of Geneious v5.6.4 (some alignments are provided as **Supplementary Images 1** and **2**). Conserved regions and potential markers were identified visually. To achieve HRM identification of the species, primers surrounding the selected markers were designed using primer 3^1^ with an estimated melting temperature of 60°C (**Table 2**). According to the tested species, the designed primers surround fragments of 315–463 bp for *trn*L and 345–389 bp for *rbc*L.

### High Resolution Melting Analysis

HRM reactions were performed on 5 μl of single seed DNA extract, in a total volume of 20 μl, using the MeltDoctor Master mix (Life technologies) on a StepOnePlus instrument (Applied Biosystems) following the manufacturers recommendations. *Trn*L pseudogene was amplified using the primers trnL-Z1-F and trnL-lg-2R at the final concentration of 0.2 μM, *rbc*L by the primers rbcL-lg-1-F and rbcL-Z2-R at the final concentration of 0.15 μM (**Table 2**). PCR conditions were adjusted as follow, an initial denaturation of 10 min at 95°C, 45 cycles of 15 s at 95°C, and 1 min at 60°C, a complete denaturation of 10 s at 95°C, 1 min at 60°C, and a continuous melt rising from 60 to 90°C with 0.3% temperature increment every 15 s.

Each extract was run in duplicate, in the presence of the usual positive, negative and process controls and in the presence of reference materials used for HRM profiles analysis. One reference material is required for each HRM profile. These reference materials were previously prepared by extraction of identified seeds using the described protocol and control of the species by sequencing.

Considering the real-time amplification results, only the samples providing cycle threshold (*C*_t_) values below 35 were considered for HRM results analysis. Analysis of the melting profiles was performed using High Resolution Melt Software v3.0 (Applied Biosystems).

## Results

### DNA Extraction from Single Seeds

A simple methodology was developed to crush individual seeds between two microscopy glass slides and extract total DNA from this crushed material. For the 50 seed lots tested (**Table 1**), extractions and amplifications were performed separately from two single seeds. DNA concentration of the obtained extracts was too low to be measured using a Nanovolume spectrophotometer. For seed lots number 22, 29, 30, and 37, only one of the extracts allowed a proper amplification. For lots number 7, 12, 15, 31, and 33, respectively, harvested in 2013, 2011, 2014, 1994, and 1987, it was not possible to obtain any amplification. For these five last seed lots, single seed extraction was performed on two more seeds with the similar results. The failure of these seed batches was not species-specific associated. The viability of the different seed lots was not assessed.

### Species Identification

**FIGURE 1:**
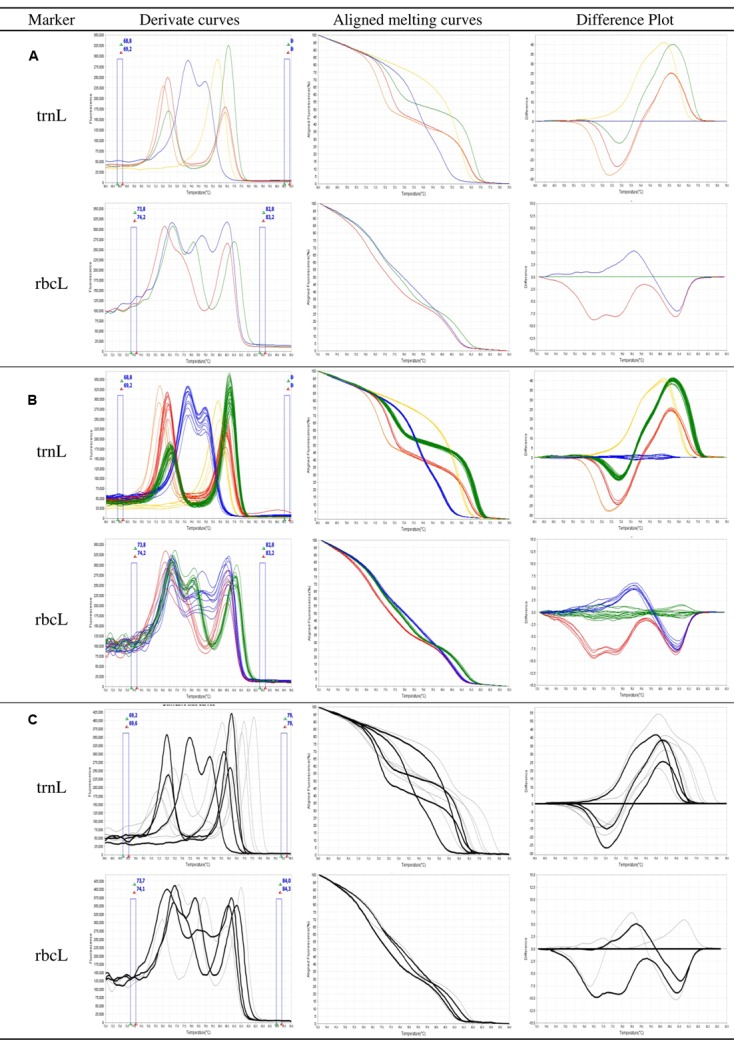
**High resolution melting (HRM) analysis of broomrape single seeds for the markers trnL and rbcL. (A)** Ability of the assay to differentiate the targeted species as different profiles (i) trnL marker: red = *Orobanche cumana*; orange = *O. cernua*; blue = *O. crenata*, *O. minor*, or *O. hederae*; green = ramosae clade; yellow = *O. foetida*; (ii) rbcL marker: red = *O. crenata*; blue = *O. minor*; green = *O. hederae*. **(B)** Consistency of the profiles between samples of a same species. **(C)** Specificity of the assay, curves in black correspond to the targeted species, while curves in gray correspond to other tested species. Derivate, aligned melting curves, and difference plots correspond to three representations of the same data: aligned melting curves have been normalized by eliminating fluorescence variance out of the melt regions; difference plots are achieved by subtracting the normalized fluorescence data of a user-defined genotype from that of each of the other samples in the HRM analysis.

For the selected markers *trn*L and *rbc*L, respectively, 9 and 11 primer pairs were designed and evaluated for their ability to provide a suitable assay. Results obtained with the best primers are reported. HRM primers were first selected according to their ability to differentiate the eight target species by providing distinct HRM profiles. Discrimination between species was possible because of sequence divergences (SNP and indel) between the amplicons. Targeted species show five different profiles when considering the high resolution melt curves of the *trn*L PCR product (**Figure 1A**). *O. cumana*, *O. cernua*, and *O. foetida* are easily identified using this HRM marker since each of these species is the only one associated with a profile (respectively, red, orange, and yellow). *O. crenata*, *O. minor*, and *O. hederae* are associated with the blue profile. They can be then differentiated from other species but not from each other. The same goes for species *P. ramosa* and *P. aegyptiaca* associated with the green profile. Among the eight species considered, the rbcL primers amplify only *O. crenata*, *O. minor*, and *O. hederae*. PCR products obtained from these three species show distinct and identifiable HRM profiles (respectively, red, blue, and green; **Figure 1A**). Considering the obtained results, an identification key is proposed to facilitate the analysis of the results (**Table 3**).

**Table 3:** Correspondence between HRM profiles obtained using the trnL and rbcL markers and broomrape species.

trnL	rbcL	Species
Red	NA	*O. cumana*
Orange	NA	*O. cernua*
Blue	Red	*O. crenata*
Blue	Blue	*O. minor*
Blue	Green	*O. hederae*
Green	NA	ramosae clade
Yellow	NA	*O. foetida*
NI	NA or NI	Unkn
NA	NA or NI	Unkn

*NA, not amplified; NI, not identified; Unkn, unknown.*

The second primers selection criterion was the consistency of the profiles between lots belonging to the same species. Amplification and HRM were performed on single seed DNA extracts obtained from the 37 (out of 50) available seed lots belonging to the eight targeted species (**Table 1**). **Figure 1B** presents the aspect of the obtained melting curve for the *trn*L and *rbc*L PCR products. Considering raw (not shown) or derivated melt curves, profiles obtained from samples of identical species show some variability. However, for identical profiles, when considering the aligned melt curves of both *trn*L and *rbc*L, the highest relative standard deviation of measured melting temperatures is 3.3%. The different profiles presented above are consistently reproduced between samples of identical species.

### Specificity of the Assay

Besides the ability of HRM primers to discriminate among weedy broomrape species, both trnL and rbcL primer pairs amplified single seed DNA extracts obtained from additional 13 seed lots initially identified based on morphological characteristics of adult plant as belonging to eight wild *Orobanche* species and three wild *Phelipanche* species (**Table 1**; **Figure 1C**). Among the 11 wild species, nine showed original non-identified profiles (NI) which could be easily distinguished from the profiles of the weedy species. Samples 38 and 39, respectively, declared in collection records as *P. purpurea* and *O. flava* were identified as *O. hederae* by HRM analysis. Samples 49 and 50 initially declared in collection records as *P. mutelii* show the same trnL profile than *P. ramosa* and *P. aegyptiaca*.

To control the identification made by HRM, sequencing of the pseudogenes *trn*L and *rbc*L was performed on all the analyzed DNA extracts. Sequences were submitted to GenBank and are available with accession numbers KX539159–KX539172. Comparison of the HRM and sequencing interpretations is presented in **Table 1**. Concerning the targeted species, results show a 100% match between interpretations obtained using both techniques. Furthermore, all the samples identified as non-target by sequencing are designated as non-identifiable by HRM. It is interesting to note that identifications performed by sequencing are not always consistent with primary identification based on visual criteria. For samples 38 and 39 visually identified as *O. purpurea* and *O. flava*, HRM identifications as *O. hederae* are consistent with sequencing results, suggesting that the HRM identification is correct and that the initial morphological identification failed. Sequencing confirmed the visual identity of the samples 49 and 50 as *P. mutelii*. HRM profile common to *P. ramosa* and *P. aegyptiaca* is therefore not specific to these two species but also includes close species.

### Reproducibility of the Assay

The HRM profiles obtained with the trnL and rbcL primers can be considered as complex. Furthermore, reading a melting profile is performed by an analyst and is somehow subjective. To question the transferability of the technique, 20 seed lots have been analyzed by three analysts in two different laboratories (GEVES and Terres Inovia). Each analyst performed the experiment on one single seed of each seed lot. Obtained results are shown in **Table 4**. For 7 out of the 20 seed lots tested, at least one extraction did not allow the amplification. When DNA was properly extracted and amplified, obtained results were in accordance except in the cases of lots 13 and 16. In one case out of three, profiles associated with these seed lots of ramosae had the correct aspect but a different melting temperature and were noted as non-identified. These differences of melting temperature were consistently reproduced, however, sequencing of trnL PCR products showed no difference of sequence between these extracts and others. Excluding the failure of proper DNA extraction on some seeds, the reproducibility of the assay (defined as the percentage of agreements between two identifications performed on a same seed lot) is of 90.9%.

**Table 4:** Data of reproducibility generated by three analysts on 20 seed lots, each analyst analyzing one single seed of each lot.

Seed lot number	Analyst			Rep.
	1	2	3	
1	ramosae clade	ramosae clade	ramosae clade	3/3
2	ramosae clade	ramosae clade	ramosae clade	3/3
3	ramosae clade	NI	ramosae clade	1/3
4	ramosae clade	ramosae clade	ramosae clade	3/3
6	NI	ramosae clade	ramosae clade	1/3
11	ramosae clade	ramosae clade	ramosae clade	3/3
13	*O. cumana*	*NA*	*O. cumana*	1/1
15	*NA*	*NA*	*O. cumana*	0/0
18	*O. cernua*	*NA*	*O. cernua*	1/1
20	*O. foetida*	*O. foetida*	*O. foetida*	3/3
21	*O. foetida*	*O. foetida*	*O. foetida*	3/3
24	*O. minor*	*O. minor*	*O. minor*	3/3
29	*O. crenata*	*O. crenata*	*O. crenata*	3/3
32	*O. crenata*	*O. crenata*	*NA*	1/1
35	*O. hederae*	*O. hederae*	*O. hederae*	3/3
37	*O. hederae*	*NA*	*O. hederae*	1/1
38	*O. hederae*	*O. hederae*	*O. hederae*	3/3
44	NI	NI	NI	3/3
46	*NA*	NI	*NA*	0/0
48	NI	NI	*NA*	1/1
				90.9%

*NA, not amplified; NI, not identified; Rep, reproducibility defined as the percentage of agreements between two identifications performed on a same seed lot (absence of amplifications have been excluded of the calculation).*

## Discussion

Seed producers may face contaminations of their crop seed lots by seeds of noxious broomrapes. In case of trading, they require international seed lot certificates provided by official seed testing stations. This is mainly carried out through analysis of specific purity of seed lots. However, if this analysis can identify seed lots containing broomrape seeds, it cannot allow a clear identification of the parasite species. Indeed, broomrape species identification can be achieved thanks to seed coat morphological features observed under microscopy (Joel, 1987; Abu Sbaih and Jury, 1994), but this approach is extremely difficult even for trained botanists and requires an extensive expertise usually not available in most laboratories. Molecular markers such as ITS, ISSR, plastid genes, or RAPD were developed for identification of broomrape species, but all required large amount of seeds incompatible with an isolation of few seeds from a specific purity analysis. Thus, protocols allowing DNA extraction from broomrape single seed were developed (Portnoy et al., 1997; Osterbauer and Rehms, 2002) and used with RAPD markers (Katzir et al., 1996). Seeds of five different species could be identified using these methods: *P. aegyptiaca*, *P. ramosa*, *O. cernua*, *O. cumana*, and *O. crenata*.

The protocol developed in this study is the first work describing the application of HRM curve analysis for differentiation of broomrape species. Compared to previous technologies, the proposed protocol and markers allow to extend the identification spectrum since it was able to differentiate between eight species, the five above mentioned plus *O. foetida*, *O. hederae*, and *O. minor*. The sequences of the root parasitic plants used in this study present variation generating divergences in the HRM patterns. Deletions, insertions, and several SNPs are responsible for the differences in the observed melting curves between the different species amplicons. The two plastid genes, *rbc*L, and *trn*L, have been already used as HRM markers for identification of plants (Madesis et al., 2012; Osathanunkul et al., 2015).

By using the HRM technology and by targeting plastid sequences, it was then possible to develop a simple, reliable, and cost effective assay to identify the seven main weedy species of broomrape potentially found in crop seed lots. In addition, it allows discrimination between these weedy species and 12 species lacking agronomic interest. The high level of divergence between species in the targeted sequences provided more complex profiles than for HRM assays targeting SNP (Toi and Dwyer, 2008) or microsatellites (Mackay et al., 2008). However, in most cases, DNA extracted from single seeds allowed a proper amplification and profiles could be identified by the analyst by comparison with the reference materials introduced in each experiment. On 45 amplified samples, the assay provided results perfectly matching with sequencing. The technique was used by several analysts in two laboratories using different HRM-capable real-time PCR machine and visual analysis of the HRM profiles. In these conditions, the technique shows a reproducibility of 90%. This rate of reproducibility is higher than the one received with RAPD markers, known to be weakly reproducible when employed in different laboratories with different PCR apparatus (Jones et al., 1997). The described assay will make then reliable identification much easier for any diagnostic or research purpose. It is also a fast close tube method not requiring post-PCR manipulation such as DNA gel electrophoresis like in RAPD analysis.

However, the success of the developed assay depends on the concentration and/or quality of the extracted DNA. Indeed for some seed lots it was not possible to reach the minimal concentration and/or quality from some tested seeds or even from any of the tested seeds. HRM technique also requires homogenous DNA extract compositions among samples to compare. Composition may indeed impact the melting temperature of the amplification products. By extracting DNA from single seeds, extracts are relatively homogeneous. However, during the evaluation of the reproducibility, some profiles showed the expected melting profile but with a different melting temperature. In a seed lot, heterogeneity of the seeds may therefore occasionally be an issue for profiles comparison.

Using the identified plastid targets, it was not possible to differentiate the species of the taxonomically difficult ramosa aggregate (*P. mutelii*, *P. ramosa*, and *P. aegyptiaca*) referred to as *ramosae clade*. Further development of the assay by adding a third marker could provide the ability to differentiate species in the ramosa aggregate. If a species identification is necessary after a *ramosae clade* or a NI result, the product obtained after the HRM amplification and denaturation can be used for sequencing as any regular PCR product.

The development of an assay able to identify broomrape species from single seeds allows testing of seeds found in commercial seed lots but also identification of mature plants from the field. Broomrape seeds are indeed a material easy to collect and transport, it can be stored at room temperature for many years. For the identification of plants at early stages (before the presence of seeds), the assay can be extended to vegetative material.

## Author Contributions

MR planned and designed the research; PD and AP performed the plastid genome sequences analysis; AD performed the experiments. MR wrote the paper with the help of PD.

## Conflict of Interest Statement

The authors declare that the research was conducted in the absence of any commercial or financial relationships that could be construed as a potential conflict of interest.

## Footnotes

1 http://primer3.ut.ee/

## Supplementary Material

IMAGE 1 **Alignment of partial sequences of the trnL gene obtained by amplification using the primers trnL C (F) and trnL HRM R of total DNA extracted from individual seeds belonging to the 8 species of interest**.

IMAGE 2 **Alignment of partial sequences of the rbcL gene obtained by amplification of total DNA extracted from individual seeds belonging to 7 of the 8 species of interest**.
