# Supplementary figures and images for: Molecular Identification of Broomrape Species from a Single Seed by High Resolution Melting Analysis
Source: Front Plant Sci. 2016 Dec 12;7:1838. doi: 10.3389/fpls.2016.01838 (PMC5149549; doi:10.3389/fpls.2016.01838)

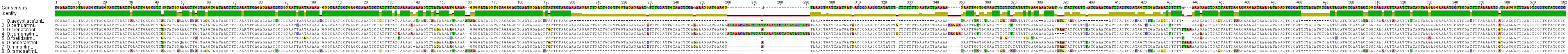

Supplement: IMAGE 1 — Alignment of partial sequences of the trnL gene obtained by amplification using the primers trnL C (F) and trnL HRM R of total DNA extracted from individual seeds belonging to the 8 species of interest. [file Image_1.JPEG]

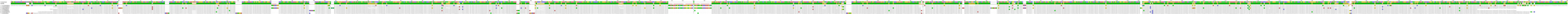

Supplement: IMAGE 2 — Alignment of partial sequences of the rbcL gene obtained by amplification of total DNA extracted from individual seeds belonging to 7 of the 8 species of interest. [file Image_2.JPEG]
